# Statistics of Mammary Cancer

**Published:** 1881-04

**Authors:** 


### EXCERPTA.

Statistics of Mammary Cancer.—The immense material accumulated at the Vienna Pathological Institute has been utilized by Torok and Wittelshofer (*Archiv fur klinische Chirurgie*, vol. vxv., 1880,) in order to determine some questionable points in relation to the statistics of mammary carcinoma. In 72,000 bodies, of which autopsical records were made between the years 1817 to 1879, mammary cancer was found 366 times, or about in one-half per cent. of the cases. In about 30,000 female corpses the tumors were found in one per cent. of all cases. In perhaps eight of them sarcoma may have been present. Of 351, in which the location of the tumor was stated, 161 exhibited disease on the right side, 141 on the left, and 46 on both sides. Three times the disease was found in very old men. The age of the dead varied from twenty to ninety years; the majority of cases occurred between forty and seventy years. In 184 instances the patients had undergone operation; of this number 105 were found without metastic deposits. Of the 182 who had not been operated upon, only 41 were free from secondary nodules. Secondary local dissemination was frequently found in the adjoining integuments, muscles, glands, pleuræ. Very rarely distant organs were found to have become involved by local extension. Thus, in the pericardium, peritoneum and liver such secondary deposits were seen in only two cases of each. Secondary metastatic neoplasms, however, were frequent. In the lymph-glands they were found 192 times, in the respiratory organs 132, in the organs of digestion 139; of which number 127 belonged to the liver.—*Centralbl. fur Chir.*, December 18, 1880.

